# Graph theory for analyzing pair-wise data: application to geophysical model parameters estimated from interferometric synthetic aperture radar data at Okmok volcano, Alaska

**DOI:** 10.1007/s00190-016-0934-5

**Published:** 2016-07-09

**Authors:** Elena C. Reinisch, Michael Cardiff, Kurt L. Feigl

**Affiliations:** Department of Geoscience, University of Wisconsin-Madison, 1215 West Dayton Street, Madison, WI 53706 USA

**Keywords:** Remote sensing of volcanoes, Numerical solutions, Transient deformation, Inverse theory

## Abstract

Graph theory is useful for analyzing time-dependent model parameters estimated from interferometric synthetic aperture radar (InSAR) data in the temporal domain. Plotting acquisition dates (epochs) as vertices and pair-wise interferometric combinations as edges defines an incidence graph. The edge-vertex incidence matrix and the normalized edge Laplacian matrix are factors in the covariance matrix for the pair-wise data. Using empirical measures of residual scatter in the pair-wise observations, we estimate the relative variance at each epoch by inverting the covariance of the pair-wise data. We evaluate the rank deficiency of the corresponding least-squares problem via the edge-vertex incidence matrix. We implement our method in a MATLAB software package called GraphTreeTA available on GitHub (https://github.com/feigl/gipht). We apply temporal adjustment to the data set described in Lu et al. (Geophys Res Solid Earth 110, 2005) at Okmok volcano, Alaska, which erupted most recently in 1997 and 2008. The data set contains 44 differential volumetric changes and uncertainties estimated from interferograms between 1997 and 2004. Estimates show that approximately half of the magma volume lost during the 1997 eruption was recovered by the summer of 2003. Between June 2002 and September 2003, the estimated rate of volumetric increase is $(6.2 \, \pm \, 0.6) \times 10^6~\mathrm{m}^3/\mathrm{year}$. Our preferred model provides a reasonable fit that is compatible with viscoelastic relaxation in the five years following the 1997 eruption. Although we demonstrate the approach using volumetric rates of change, our formulation in terms of incidence graphs applies to any quantity derived from pair-wise differences, such as range change, range gradient, or atmospheric delay.

**Electronic supplementary material:**

The online version of this article (doi:10.1007/s00190-016-0934-5) contains supplementary material, which is available to authorized users.

## Introduction

### Background of InSAR

Interferometric synthetic aperture radar (InSAR) measures the deformation of an area on the ground by calculating the difference in phase between two synthetic aperture radar (SAR) images covering the same location taken at two different points in time (“epochs”) (e.g., Massonnet and Feigl 1998). Like many geodetic techniques, including spirit leveling, triangulation by theodolite, trilateration by electronic distance measurement (EDM), and very long baseline interferometry (VLBI), InSAR makes relative measurements as pair-wise differences (e.g., Feigl 2002). Each pixel in an unwrapped interferogram is the difference between the satellite-to-ground range measured at one epoch and the range measured at a second epoch (e.g., Massonnet and Feigl 1998).

Using the notation conventionally employed in geophysics, we write a linear model as $\mathbf {Gm} = \mathbf {d}$, where $\mathbf {d}$ is a vector containing *n* pair-wise measurements, $\mathbf {m}$ is a vector containing *m* parameters in the geophysical model, and $\mathbf {G}$ is an *n*-by-*m* design matrix (e.g., Aster et al. 2013). In the case of pair-wise data, the design matrix $\mathbf {G}$ is an incidence matrix consisting of elements from the set $\{-1,0,1\}$ (Strang and Borre 1997). Alternatively, Schmidt (1996) expresses differences in range in terms of bivectors to estimate relative position coordinates. To estimate the optimal set of parameters, one conventionally solves the (weighted) least-squares problem. If the geophysical model is a function of time, we call the estimation procedure “temporal adjustment” (Feigl 2002; Berardino et al. 2002; Beauducel et al. 2000; Schmidt and Bürgmann 2003).

In the case of InSAR, the input data can consist of: (1) a set of differential changes in range along the line of sight at a single pixel, or (2) a set of model parameters estimated from individual interferometric pairs spanning different intervals of time. In the example application that we consider below, the model parameters measure the volumetric change in a magma chamber below the volcanic edifice. With such data, the number of epochs *q* is necessarily greater than the minimum number of pair-wise combinations $c = q - 1$ required to span the epochs (Feigl and Thurber 2009). Consequently, if the number of model parameters *m* equals the number of epochs *q*, then the corresponding least-squares problem is necessarily underdetermined. In this case, the design matrix $\mathbf {G}$ is rank-deficient.

For example, suppose we have InSAR data acquired by a radar sensor measuring an inflating volcano. In this case, one pair-wise observation will measure the change in range (distance along the line of “sight” from the sensor to the ground) between the first and second epochs. The decrease in range equals the increase in relative displacement along the line of sight of the sensor (i.e., the temporal change in a single component of relative position). Without additional constraining information, such as the initial position with respect to a known reference point, we cannot estimate absolute parameters. To solve the least-squares problem, we must reduce the number of parameters and/or add regularizing constraints.

In this paper, we apply graph theory to:

1. visualize model parameters estimated from an InSAR data set,
2. construct the covariance matrix for pair-wise data,
3. estimate the error variance of the measurements at each epoch,
4. evaluate the rank deficiency of the least-squares inverse problem,
5. select appropriate parameterizations of the time-dependent model, and
6. select regularizing constraints.

We have included a table describing our mathematical notation in Online Resource 1.

### Previous work

**Fig. 1:**
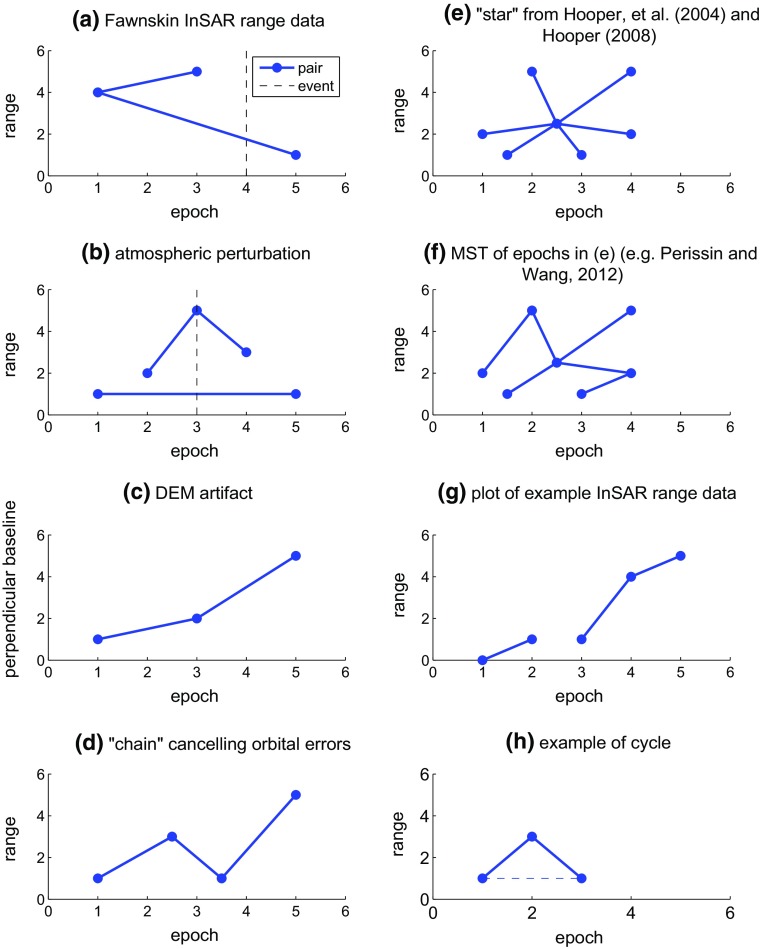
Examples of common graphs in InSAR analysis. In each graph, the *dots* correspond to epochs (vertices) and *segments* correspond to pairs (edges). **a** Range changes depend on whether or not the measured interval of time spans an event such as the Fawnskin earthquake (Feigl et al. 1995; Feigl and Thurber 2009). **b** Atmospheric perturbation. The atmospheric perturbation at epoch 3 creates a significant disturbance in pairs (2, 3) and (3, 4) but not (1, 5). **c** An error in the digital elevation model (DEM) can create an artifact in the interferogram that is proportional to the orbital separation (Massonnet and Feigl 1995), also known as the “baseline distance B” (Zebker and Goldstein 1986). **d** A graph of a “chain”, where the chronologically first epoch of one pair is the chronologically second epoch of the previous pair (Biggs et al. 2007). The orbital errors associated with the interior epochs in the chain cancel each other. **e** The graph of a data set that forms a “star” such that each of the second epochs per pair forms a pair with the single master epoch located at the center (e.g., Hooper et al. 2004; Hooper 2008). **f** Example of the minimum spanning tree (MST) of the same data set as in **e** (e.g., Perissin and Wang 2012). **g** Incidence graph of example data set containing 5 epochs, 3 pairs, and 2 distinct trees. **h** Three epochs form only two independent pairs (*solid line* segments). Adding a third pair (*dashed line* segment) forms a cycle in the graph, but adds no additional information to the inverse problem. The information gained from the combination of pairs (1, 2) and (2, 3) is the same as the information given from pair (1, 3)

To distinguish between geophysical signals on the ground, perturbations in the atmosphere, and artifacts in the processing, one can compare different interferometric pairs that span different time intervals, as sketched in panels a through c of Fig. 1 (e.g., Massonnet and Feigl 1995). In terms of graph theory, Fig. 1a, c are examples of a tree graph, where the edges of the graph connect the vertices without any cycles. Figure 1b is an example of a collection of trees, also known as a forest (Harris et al. 2008).

Biggs et al. (2007) introduce the notion of “chains”, as sketched in Fig. 1d. Constructing a chain of pairs where the second epoch of one pair is the first epoch of the next pair cancels all of the atmospheric contributions except those of the first and last epochs in the chain. In terms of graph theory, a “chain” is a path graph, or a tree with no branches connecting all vertices, such that it has internal nodes of degree two and terminal nodes of degree one [see Fig. 1d (e.g., Harris et al. 2008, pg. 6)].

Alternatively, the single-master approach refers all the pairs to a single epoch in a graph that resembles a star (Fig. 1e) (e.g., Hooper et al. 2004; Hooper 2008). Perissin and Wang (2012) draw the graph of a minimum spanning tree (Fig. 1f) in two dimensions: time and orbital separation. A minimum spanning tree is a tree which contains all vertices of a graph and has the lowest cost, calculated over all edges (e.g., Harris et al. 2008, pg. 39).

Alternatively, one can choose a set of pairs such that the time intervals between successive epochs and the orbital separations (“baselines”) between pairs are as short as possible in an approach known as Small Baseline Subset (SBAS) (Berardino et al. 2002; Lanari et al. 2007; Casu et al. 2008; Lee et al. 2012).

In a different approach, Hetland et al. (2012) generalize the temporal parameterization to include a library of temporal functions in their Multiscale InSAR Time Series (MInTS) procedure. More recently, Agram and Simons (2015) have developed a model for spatial and temporal covariance for interferometric phase noise for use in time-series analysis.

## Review of graph theory

A graph represents the relationships between a set containing vertices and a set of edges (Harris et al. 2008). When applied to InSAR, the *m* vertices signify *points* in time, or epochs, and the edges signify the *n* pair-wise combinations of images, or interferograms that span *intervals* of time. Following the notation of Merris (1994), we draw a directed graph by assigning a direction to each edge, with one end of the pair assigned as the “positive” vertex and the other as the “negative” vertex. For example, given a vertex $v_j$ at an epoch $t_j$ and another vertex $v_k$ at a second, chronologically later, epoch $t_k$, we denote the $i{\text {th}}$ pair as edge $e_{i}=\{v_{j},v_{k}\}$. The resulting edge-vertex incidence matrix $\mathbf {Q}$ has *n* rows and *m* columns. The $i{\text {th}}$ row at $\mathbf {Q}$ represents the $i{\text {th}}$ pair such that $Q_{i,j} = -1$, $Q_{i,k} = 1$, and $Q_{i,v \notin \{j,k\}} = 0$ for all other vertices other than $v_j$ and $v_k$. Note that our edge-vertex incidence matrix $\mathbf {Q}$ is the transpose of the *m*-by-*n* vertex-edge incidence matrix used by Merris (1994). Subsequently, we use the edge-vertex form of the incidence matrix $\mathbf {Q}$ to lighten the notation.

### Relationship between components and undetermined parameters

We consider a situation where InSAR data corresponding to range changes $\mathbf {\Delta } \varvec{\rho }$ from a set of *n* interferometric pairs are derived from distinct SAR images acquired at *m* epochs. The corresponding system of linear equations is $\mathbf {Q} \mathbf {m} = \mathbf {d}_\mathrm{obs}$, where $\mathbf {d}_\mathrm{obs} = \mathbf {\Delta } \varvec{\rho }$ is the vector of pair-wise observations and $\mathbf {m} = \varvec{\rho }$ is the vector of *m* unknown parameters, each of which represents the absolute range from sensor to target at an epoch in time. The solution is underdetermined because the number of unknown parameters *m* is greater than the number of independent equations $\mathrm{rank}(\mathbf {Q})$. The rank deficiency, or number of undetermined parameters, is

1 $$\begin{aligned} \mu = m-\mathrm{rank}(\mathbf {Q}) \end{aligned}$$

as demonstrated by Strang and Borre (1997), p. 114, 118.

In some cases, the graph is disconnected, or composed of more than one distinct component [also referred to as a connected component (e.g., Harris et al. 2008, p. 8)]. Feigl and Thurber (2009) called each component a “species.” A distinct component in an InSAR data set is a set of individual images that combine pair-wise to form a set of interferograms. The epoch of each image corresponds to a vertex in the graph. Each vertex in a component is connected to at least one other vertex in the component by an edge. One distinct component is not connected to another distinct component. For example, SAR images acquired by one radar sensor are not interferometrically compatible with those from another sensor. The vertices in the corresponding graph thus form two distinct, disconnected components, as sketched in Fig. 1g.

Graph theory tells us that the rank of the incidence matrix for a disconnected graph is

2 $$\begin{aligned} \mathrm{rank}(\mathbf {Q}) = m - k, \end{aligned}$$

where *m*, the number of nodes (or vertices), represents the number of epochs and *k* is the number of components (Deo 2004, p. 140). If the components do not contain any cycles, then *k* also represents the number of distinct trees. Thus, the number of components in a disconnected graph is equal to the rank deficiency of the corresponding incidence matrix.

#### Theorem 1

3 $$\begin{aligned} k = m - \mathrm{rank}(\mathbf {Q}) = \mu \end{aligned}$$

Feigl and Thurber (2009) note that the number of distinct components in the graph of InSAR pairs equals the rank deficiency of the incidence matrix. Grossman et al. (1995) prove that this equality follows directly from graph theory in their Theorem 2.5.

## Methods

### Example case: building the design matrix $\mathbf {G}$

Let us assume a simple, specific case, as graphed in Fig. 1g, with five epochs $\{v_{1},v_{2},v_{3},v_{4},v_{5}\}$ and three pairs $\{e_{1,2},e_{3,4},e_{4,5}\}$. The corresponding graph is disconnected (Fig. 1g). It includes two distinct components. The first component includes 1 pair and 2 epochs. The second component includes 2 pairs and 3 epochs. Since there are no cycles, each of these two components is a tree. Since the epochs are arranged in chronological order, the graph is directed. Thus, it is a directed acyclic graph (DAG).

The corresponding edge-vertex incidence matrix $\mathbf {Q}$ is:

4 $$\begin{aligned} \mathbf {Q}=\begin{bmatrix} -1&\quad 1&\quad 0&\quad 0&\quad 0 \\ 0&\quad 0&\quad -1&\quad 1&\quad 0 \\ 0&\quad 0&\quad 0&\quad -1&\quad 1 \end{bmatrix} \end{aligned}$$

The system of equations is thus

5 $$\begin{aligned} \begin{bmatrix} -1&\quad 1&\quad 0&\quad 0&\quad 0 \\ 0&\quad 0&\quad -1&\quad 1&\quad 0 \\ 0&\quad 0&\quad 0&\quad -1&\quad 1 \end{bmatrix} \begin{bmatrix} m_1 \\ m_2 \\ m_3 \\ m_4 \\ m_5 \end{bmatrix}&= \begin{bmatrix} d_{1,2} \\ d_{3,4} \\ d_{4,5} \end{bmatrix} \nonumber \\&= \begin{bmatrix} \rho _2 - \rho _1 \\ \rho _4 - \rho _3 \\ \rho _5 - \rho _4 \\ \end{bmatrix}\nonumber \\ \end{aligned}$$

where $\rho _i = \rho (t_i)$ is the range at epoch $t_i$. Using Fig. 1g, Theorem 1, and Eq. (3), we see that since $\mu =k=2$, two parameters cannot be determined. Thus, we need to add two constraints to the system in order to regularize the problem. We append two constraining rows to $\mathbf {Q}$ to formulate the design matrix $\mathbf {G}$ for the constrained system $\mathbf {Gm}=\mathbf {d}$. From Fig. 1g, we see that the two initial vertices of interest are $v_{1}$ and $v_{3}$, corresponding to epochs $t_1$ and $t_3$.

For simplicity, let us (arbitrarily) constrain the system such that the absolute range at the first epoch in each distinct component is fixed at zero.

6 $$\begin{aligned} \rho (t_1)&= m_1 = 0 \nonumber \\ \rho (t_3)&= m_3 = 0 \end{aligned}$$

From these equations, we define a 2-row constraint matrix $\mathbf {C}$:

7 $$\begin{aligned} \mathbf {C} = \begin{bmatrix} 1&\quad 0&\quad 0&\quad 0&\quad 0 \\ 0&\quad 0&\quad 1&\quad 0&\quad 0 \end{bmatrix} \end{aligned}$$

and a 2-row data constraint vector: $\mathbf {d}_\mathrm{con} = {[0, 0]}^\intercal$.

**Fig. 2:**
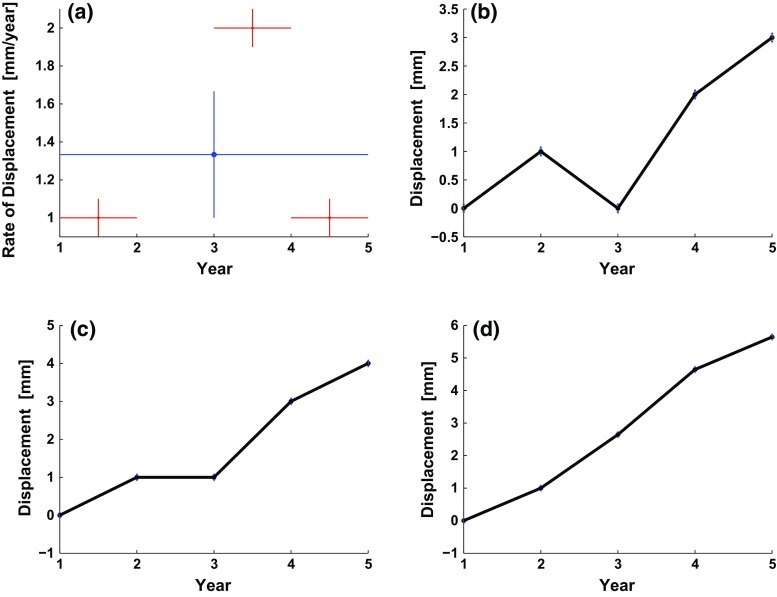
*Plots* illustrating the simple, fictitious example of vertical displacement on an inflating volcano as measured by an InSAR data set with 5 epochs and 3 independent pairs for which the graph contains two distinct trees. **a** *Plot* of displacement rates for individual pairs with corresponding time intervals (*horizontal red bars*), and standard deviations of measurement errors (*vertical red bars*). The *blue symbol* indicates weighted mean with scaled 68 % confidence interval (*vertical blue bar*) and time span for the entire data set (*horizontal blue bar*). **b** *Plot* of displacement as a function of time as calculated by temporal adjustment using a piecewise linear parameterization and two constraints. The constraining equations plot as relative displacement within each tree, setting the initial displacement at the first epoch in each distinct component at zero. **c** *Plot* of displacement as a function of time estimated with a piecewise linear parameterization in terms of velocity using the method outlined in Berardino et al. (2002). **d** *Plot* of displacement as a function of time estimated using the parameterization in part *c* with first-order Tikhonov regularization (favoring a constant-rate solution). The regularization parameter $\beta = 0.0090$

Here, the number of rows in each of $\mathbf {C}$ and $\mathbf {d}_\mathrm{con}$ is equal to the number of constraints $k=2$. We construct the design matrix $\mathbf {G}$ from the incidence matrix $\mathbf {Q}$ and the constraint matrix $\mathbf {C}$:

8 $$\begin{aligned} \mathbf {G}=\begin{bmatrix} \mathbf {Q} \\ \mathbf {C} \end{bmatrix}. \end{aligned}$$

We expand the data vector by including the constraining elements of Eq. (6) in the data vector $\mathbf {d}$.

9 $$\begin{aligned} \mathbf {d}= \begin{bmatrix} \mathbf {d}_\mathrm{obs} \\ \mathbf {d}_\mathrm{con} \end{bmatrix} . \end{aligned}$$

Now the system takes the form

10 $$\begin{aligned} \begin{bmatrix} \mathbf {Q} \\ \mathbf {C} \end{bmatrix} \begin{bmatrix} \mathbf {m} \end{bmatrix} = \begin{bmatrix} \mathbf {d}_\mathrm{obs} \\ \mathbf {d}_\mathrm{con} \end{bmatrix}. \end{aligned}$$

Let us further define a simple data set of range changes $\mathbf {d}_\mathrm{obs} = {[1, 2, 1]}^\intercal (\mathrm {mm})$ and a set of epochs at 1-year intervals, $\mathbf {t} = {[1, 2, 3, 4, 5]}^\intercal (\mathrm {years})$. Figure 2a shows the data set as rates. Figure 2b shows how adding constraints leads to a solution.

### Alternative parameterization

An alternative parameterization is the method of rates developed by Berardino et al. (2002). This method chooses a vector $\mathbf {m} = \mathbf {v}$ of rate parameters such that each element $v_i$ represents the rate of change in displacement (i.e., velocity) between each pair of consecutive epochs (i.e., during the $i{\text {th}}$ interval of time). Given a set of *n* pairs and a vector $\mathbf {t}$ of *m* epochs, we solve for a vector $\mathbf {v}$ of $m-1$ rate parameters using the Berardino design matrix $\mathbf {G^{\text {[B]}}}$ via the following procedure.

*Step 1* Define an edge-vertex matrix $\mathbf {\Delta }$ with $m-1$ rows and *m* columns representing the edge-vertex incidence matrix corresponding to a path graph chronologically connecting all epochs in the data set. In our 2-component example, the path connects the two distinct components.

*Step 2* Find the pair-rate matrix $\mathbf {B}$, where the $i,j{\text {th}}$ element $B_{i,j} = 1$ if the $i{\text {th}}$ pair corresponds to the $j{\text {th}}$ rate and $B_{i,j} = 0$ otherwise. This matrix can be calculated from the product of the incidence matrix $\mathbf {Q}$ and the pseudoinverse $\mathbf {\Delta }^{\dag }$ of the edge-vertex matrix:

11 $$\begin{aligned} \mathbf {B} = \mathbf {Q} \mathbf {\Delta }^{\dag } . \end{aligned}$$

Here, we note that $\mathbf {B}$ is an incidence matrix denoting the relationship between pairs and rates.

*Step 3* Define a diagonal $(m-1)$-by-$(m-1)$ matrix $\mathbf {T}$ with the time intervals between consecutive epochs as the diagonal elements and zeros as the off-diagonal elements:

12 $$\begin{aligned} T_{i,j} = \delta _{i,j} (t_{i+1} - t_{i}) \end{aligned}$$

where $\delta _{i,j}$ is the Kronecker delta

13 $$\begin{aligned} \delta _{i,j} = {\left\{ \begin{array}{ll} 0 &{} \text {if } i \ne j \\ 1 &{} \text {if } i = j \\ \end{array}\right. } \end{aligned}$$

*Step 4* Find the Berardino design matrix $\mathbf {G}_{n\times (m-1)}^{\text {[B]}}$ from the product of the pair-rate incidence matrix $\mathbf {B}$ and the diagonal matrix of time intervals $\mathbf {T}$:

14 $$\begin{aligned} \mathbf {G}_{n\times (m-1)}^{[\text {B}]} = \mathbf {B}\mathbf {T} \end{aligned}$$

*Step 5* Estimate the vector of rate parameters $\mathbf {v}$ by solving $\mathbf {G}^{[\text {B}]} \mathbf {v} = \mathbf {d}$ using singular value decomposition (SVD).

We note that this method fails when the data set includes more than one distinct tree because the system of equations is rank deficient. Thus, the minimum-norm solution given by singular value decomposition will tend to oscillate. For example, we consider again the case graphed in Fig. 1g with $n=3$ distinct pairs of displacements $\mathbf {d} = \{d_{1,2}, d_{3,4}, d_{4,5}\}$. We represent the epochs in chronological order as a vector $\mathbf {t} = \{t_1, t_2, t_3, t_4, t_5\}$. The data provide no information regarding the velocity $v_{2,3}$ during the interval between $t_2$ and $t_3$. We begin by defining the edge-vertex matrix $\mathbf {\Delta }$ for the path connecting all epochs:

15 $$\begin{aligned} \mathbf {\Delta }=\begin{bmatrix} -1&\quad 1&\quad 0&\quad 0&\quad 0 \\ 0&\quad -1&\quad 1&\quad 0&\quad 0 \\ 0&\quad 0&\quad -1&\quad 1&\quad 0 \\ 0&\quad 0&\quad 0&\quad -1&\quad 1 \end{bmatrix}. \end{aligned}$$

We next solve for the pair-rate incidence matrix $\mathbf {B}$ by Eq. (11) using the pseudoinverse $\mathbf {\Delta }^{\dag }$ and the incidence matrix $\mathbf {Q}$ from Eq. (4)

16 $$\begin{aligned} \mathbf {B}&= \begin{bmatrix} 1&\quad 0&\quad 0&\quad 0 \\ 0&\quad 0&\quad 1&\quad 0 \\ 0&\quad 0&\quad 0&\quad 1 \end{bmatrix}. \end{aligned}$$

We write the diagonal time-interval matrix $\mathbf {T}$ according to Eq. (12) as

17 $$\begin{aligned} \mathbf {T} = \begin{bmatrix} (t_2 - t_1)&\quad 0&\quad 0&\quad 0 \\ 0&\quad (t_3-t_2)&\quad 0&\quad 0 \\ 0&\quad 0&\quad (t_4-t_3)&\quad 0 \\ 0&\quad 0&\quad 0&\quad (t_5-t_4) \end{bmatrix}. \end{aligned}$$

Finally, we solve for $\mathbf {G}^{[\text {B}]}$ using Eq. (14):

18 $$\begin{aligned} \mathbf {G}^{[\text {B}]}&= \mathbf {BT} \nonumber \\&= \begin{bmatrix} (t_2-t_1)&\quad 0&\quad 0&\quad 0 \\ 0&\quad 0&\quad (t_4-t_3)&\quad 0 \\ 0&\quad 0&\quad 0&\quad (t_5-t_4) \end{bmatrix}. \end{aligned}$$

The system of equations now takes the form $\mathbf {G}^{[\text {B}]}\mathbf {v} = \mathbf {d}_\mathrm{obs}$.

19 $$\begin{aligned} \begin{bmatrix} (t_2-t_1)&\quad 0&\quad 0&\quad 0 \\ 0&\quad 0&\quad (t_4-t_3)&\quad 0 \\ 0&\quad 0&\quad 0&\quad (t_5-t_4) \end{bmatrix} \begin{bmatrix} v_{1,2} \\ v_{2,3} \\ v_{3,4} \\ v_{4,5} \end{bmatrix}&= \begin{bmatrix} d_{1,2} \\ d_{3,4} \\ d_{4,5} \end{bmatrix} \end{aligned}$$

where $\mathbf {v}$ is the parameter vector of velocities and $\mathbf {d}_\mathrm{obs}$ is the vector of observed pair-wise, relative displacements. The design matrix $\mathbf {G}^{[\text {B}]}$ has a rank deficiency of 1. Its null column indicates a lack of information during the time interval between epochs $t_2$ and $t_3$. Consequently, the relative velocity $v_{2,3}$ between the two distinct trees (disconnected components) of the incidence graph (Fig. 2c) is unconstrained. In other words, the path graph described by $\mathbf {\Delta }$ includes an edge $e_{2,3}$ that is not in the 2-component graph described by $\mathbf {Q}$.

Singular value decomposition gives a minimum-norm solution, assuming no movement where there is a lack of information. In general, the minimum-norm solution by SVD becomes more prone to local oscillations as the number of unconstrained parameters increases (Aster et al. 2013, pp. 75, 93). To alleviate the locally oscillatory nature of the minimum-length solution found through SVD of Berardino et al. (2002)’s method, we consider other regularizations. We recognize that the minimum-length least-squares solution of this method found from SVD is equivalent to the zeroth-order Tikhonov solution of Berardino et al. (2002) in the limit as the regularization parameter approaches zero.

Instead, we choose to impose first-order Tikhonov regularization using an $(m-1)$-by-*m* matrix $\mathbf {W}$ that quantifies the roughness of the solution and a regularization parameter $\beta$ (e.g., Aster et al. 2013). The purpose of the first-order roughening matrix $\mathbf {W}$ is to favor constant-rate solutions. The regularization parameter $\beta$ allows us to choose how much we favor the minimum misfit over our desire for a constant-rate solution (and vice versa). To resolve this trade-off, we use an L-curve to compare the L2 norms of the model vectors with those of the residual vectors. Figure 2d shows an application of first-order Tikhonov regularization to the Berardino et al. (2002) parameterization of our example case with the regularization parameter set to $\beta = 0.0090$. The regularized solution tends towards constant rates. We describe a practical example in Sect. 4.6 below.

### Design matrix $\mathbf {G}$ for other functions of time

Hetland et al. (2012) develop a “library” of functions to describe the time dependence of InSAR data. Following their approach, we formulate the design matrix $\mathbf {G}$ to represent temporal functions that are more complicated than the piecewise-linear polynomials just presented. In these cases, we formulate our system of equations to represent the product of a vector of model parameters $\mathbf {m}$ and a temporal function *f*(*t*). This parameterization assumes that the time-dependent and spatial-dependent functions are separable. Thus, we consider only the time-dependent function in our analysis. We write an element of the design matrix $\mathbf {G}$ corresponding to the $j{\text {th}}$ element of the model parameter $m_j$

20 $$\begin{aligned} G_{i,j} = f_j(t_{2,i}) - f_j(t_{1,i}) \end{aligned}$$

where $t_1$ and $t_2$ refer to the first and second epochs of the $i{\text {th}}$ pair, respectively. Now we have a system of equations $\mathbf {Gm} = \mathbf {d}$ where $\mathbf {m}$ is the vector of unknown parameters and $\mathbf {d}$ is the vector of pair-wise data. If we choose the temporal function *f*(*t*) wisely to reduce the number of parameters such that $\mathbf {G}$ has full column rank, then constraints are not necessary to solve the least-squares problem. The parameter vector $\mathbf {m}$ contains *m* elements indexed $m_i$. For example, we use a temporal function with a single parameter $m_1$

21 $$\begin{aligned} mf_{\text {exp}}(t_i) = m_1 \left( 1 - \text {exp} \left[ -\frac{t_i-t_{q}}{\tau _{m}}\right] \right) \end{aligned}$$

to model our data set, where $t_i$ is the $j{\text {th}}$ epoch, $t_q$ is a predefined reference epoch and $\tau _m$ is a predefined characteristic time scale. In practice, the values of parameters such as $t_q$ and $\tau _m$ may not be known. In this case, we use nonlinear optimization in conjunction with the linear inversion problem to choose the best-fitting values of the parameters based on the residual misfit to the data, as described below in Sect. 4.7.

### Defining the data covariance matrix

To account for the measurement uncertainty in the temporal dimension of the observed data $\mathbf {d}$, we construct the data covariance matrix $\mathbf {\Sigma }_{d}$. In the case of pair-wise InSAR data (unwrapped interferograms), the observed values of range change are given for the edges. We can employ what Merris (1994) calls the *n*-by-*n* edge-version of the Laplacian matrix

22 $$\begin{aligned} \mathbf {K} = \mathbf {Q}\mathbf {Q}^{\intercal }, \end{aligned}$$

where $\mathbf {Q}$ again represents our edge-vertex incidence matrix.

Spielman (2010) defines the normalized edge Laplacian as

23 $$\begin{aligned} \mathbf {L} = \mathbf {D}^{-1/2}\,\mathbf {K}\,\mathbf {D}^{-1/2} \end{aligned}$$

where the edge-degree matrix $\mathbf {D}$ is an *n*-by-*n* square matrix with the degrees of the edges on the diagonal and zeros elsewhere (Harris et al. 2008). [Note that our $\mathbf {D}$ is not the same as the incidence matrix denoted by the same symbol in Feigl and Thurber (2009).]

Each of the diagonal elements in the normalized edge Laplacian $\mathbf {L}$ is unity. The off-diagonal elements are $\pm \frac{1}{2}$ for pairs sharing a common epoch and zero elsewhere. Thus, the matrix $\mathbf {L}$ is the data correlation matrix (Merris 1994). It is similar to the correlation matrix for a triangulation network composed of angles (differences of directions) measured by theodolite (e.g., Prescott 1976). The off-diagonal elements of this data correlation matrix account for the temporal correlation between interferometric pairs sharing a common epoch. Two pairs of data have a correlation coefficient of $+\frac{1}{2}$ if they share a common first or second epoch, $-\frac{1}{2}$ if they share a common epoch but the epoch is first in one pair and second in the other, and zero otherwise. The correlation matrix $\mathbf {L}$ leads to the corresponding *n*-by-*n* covariance matrix for pair-wise data:

24 $$\begin{aligned} \mathbf {\Sigma }_{d} = \mathbf {S}\,\mathbf {L}\,\mathbf {S}, \end{aligned}$$

where $\mathbf {S}$ is a diagonal matrix containing the standard deviation *s* of each measured pair.

This result derived from graph theory validates formulae presented by Hanssen (2001) in his equation (3.1.4). It also reformulates the results derived by Emardson et al. (2003) in their equation (31), Biggs et al. (2007) in their equation (5), and Agram and Simons (2015) in their equation (10). This graphical formulation of the data covariance matrix is necessary to derive the covariance matrix of relative epoch-wise errors, which is new to InSAR and described in further detail in Sect. 3.6.

### Example case: data covariance matrix

Let us resume the example depicted in Fig. 1g, containing five epochs $\{t_{1},t_{2},t_{3},t_{4},t_{5}\}$ corresponding to five vertices and $n=3$ pairs $\{e_{1,2},e_{3,4},e_{4,5}\}$ in $k=2$ distinct trees. Given the incidence matrix **Q** (in Eq. (4)), we first calculate the 3-by-3 edge Laplacian using Eq. (22).

25 $$\begin{aligned} \mathbf {K}&= \mathbf {Q}\mathbf {Q}^{\intercal } \nonumber \\&= \begin{bmatrix} 2&\quad 0&\quad 0 \\ 0&\quad 2&\quad -1 \\ 0&\quad -1&\quad 2 \end{bmatrix}. \end{aligned}$$

To write the normalized edge Laplacian $\mathbf {L}$, we also need the edge-degree matrix $\mathbf {D}$. We sum the absolute values of the elements in each row of the edge-vertex incidence matrix $\mathbf {Q}$ and diagonalize the result into a 3-by-3 matrix:

26 $$\begin{aligned} \mathbf {D} = \begin{bmatrix} 2&\quad 0&\quad 0 \\ 0&\quad 2&\quad 0 \\ 0&\quad 0&\quad 2 \end{bmatrix} \end{aligned}$$

From Eq. (23), the correlation matrix is:

27 $$\begin{aligned} \mathbf {L}&= \mathbf {D}^{-\frac{1}{2}}\,\mathbf {K}\,\mathbf {D}^{-\frac{1}{2}} \nonumber \\&= \begin{bmatrix} 1&\quad 0&\quad 0 \\ 0&\quad 1&\quad -\frac{1}{2} \\ 0&\quad -\frac{1}{2}&\quad 1 \end{bmatrix} \end{aligned}$$

Finally, we use Eq. (24) to find the data covariance matrix $\mathbf {\Sigma }_{d}$. Writing the pair-wise measurement errors as a diagonal matrix $\mathbf {S}=\text {diag}({s}_{1,2},{s}_{3,4},{s}_{4,5})$, we find the covariance of the pair-wise data

28 $$\begin{aligned} \mathbf {\Sigma }_{d}&= \begin{bmatrix} {s}_{1,2}^{2}&\quad 0&\quad 0 \\ 0&\quad {s}_{3,4}^{2}&\quad -\frac{{s}_{3,4}{s}_{4,5}}{2} \\ 0&\quad -\frac{{s}_{3,4}{s}_{4,5}}{2}&\quad {s}_{4,5}^{2} \end{bmatrix}. \end{aligned}$$

The data covariance matrix $\mathbf {\Sigma }_{d}$ can be inverted because a Laplacian matrix is necessarily positive semi-definite (Merris 1994).

### Epoch-wise covariance

In the previous section, we introduced a formulation for the covariance matrix of (pair-wise) data. Now we use that formulation to estimate the relative covariance matrix for epoch-wise measurements. Given the edge-vertex incidence matrix $\mathbf {Q}$ and assuming a covariance matrix $\mathbf {\Sigma }_{\rho }$ for the epoch-wise measurements, we can use the principle of covariance propagation (e.g., equation (2.22) of Aster et al. 2013) to write the covariance matrix $\mathbf {\Sigma }_{d}$ of the pair-wise data:

29 $$\begin{aligned} \mathbf {\Sigma }_{d} = \mathbf {Q} \mathbf {\Sigma }_{\rho } \mathbf {Q}^{\intercal } \end{aligned}$$

Setting the two expressions (24) and (29) for the pairwise covariance $\mathbf {\Sigma }_{d}$ equal to each other, we find

30 $$\begin{aligned} \mathbf {Q} \mathbf {\Sigma }_{\rho } \mathbf {Q}^{\intercal } = \mathbf {SLS} \end{aligned}$$

Since neither $\mathbf {Q}$ nor $\mathbf {Q}^{\intercal }$ is a square matrix, we cannot invert them. However, we can manipulate these matrices using $\mathbf {Q}^{\intercal }$ and $\mathbf {Q}$, respectively, to rewrite Eq. (30) in terms of square matrices:

31 $$\begin{aligned} \mathbf {Q}^{\intercal } \mathbf {Q} \mathbf {\Sigma }_{\rho } \mathbf {Q}^{\intercal } \mathbf {Q} = \mathbf {Q}^{\intercal } \mathbf {\Sigma }_{d} \mathbf {Q} \end{aligned}$$

We can now multiply by the inverse of $(\mathbf {Q}^{\intercal } \mathbf {Q})$

32 $$\begin{aligned}&{(\mathbf {Q}^{\intercal } \mathbf {Q})}^{-1} (\mathbf {Q}^{\intercal } \mathbf {Q}) \mathbf {\Sigma }_{\rho } (\mathbf {Q}^{\intercal } \mathbf {Q}) {(\mathbf {Q}^{\intercal } \mathbf {Q})}^{-1} \nonumber \\&={(\mathbf {Q}^{\intercal } \mathbf {Q})}^{-1} \mathbf {Q}^{\intercal } \mathbf {\Sigma }_{d} \mathbf {Q} {(\mathbf {Q}^{\intercal } \mathbf {Q})}^{-1} \end{aligned}$$

to arrive at an equation representing the covariance of the epoch-wise measurements

33 $$\begin{aligned} \mathbf {\Sigma }_{\rho } = {(\mathbf {Q}^{\intercal } \mathbf {Q})}^{-1} \mathbf {Q}^{\intercal } \mathbf {\Sigma }_{d} \mathbf {Q} {(\mathbf {Q}^{\intercal } \mathbf {Q})}^{-1} \end{aligned}$$

Equation (33) is equivalent to the general covariance matrix of model parameters for a least-squares solution (e.g., Aster et al. 2013, p. 31). However, for the *n*-by-*m* edge-vertex incidence matrix $\mathbf {Q}$, its inverse ${(\mathbf {Q}^{\intercal } \mathbf {Q})}^{-1}$ strictly does not exist. If we regularize the incidence matrix by adding a constraint of zero mean for each component, then we can examine the relative uncertainty of the epoch-wise measurements. To do so, we include *k* more equations to the system of equations corresponding to the *k* components of the data set. The modified system of equations is

34 $$\begin{aligned} \mathbf {Q}^{\prime } \mathbf {m}= \begin{bmatrix} \mathbf {Q} \\ \mathbf {C} \end{bmatrix} \mathbf {m} = \begin{bmatrix} \mathbf {d} \\ 0 \end{bmatrix} =\mathbf {d}' \end{aligned}$$

Here, we have appended *k* zeros to the data vector $\mathbf {d}$ and a *k*-by-*m* constraint matrix $\mathbf {C}$ to the edge-vertex incidence matrix $\mathbf {Q}$. The constraint matrix $\mathbf {C}$ consists of non-zero elements $C_{i,j}= 1/\eta _i$ when *j* is the index of an epoch belonging to the $i{\text {th}}$ component. The integer $\eta _i$ is the number of epochs in the $i{\text {th}}$ component. Similarly, we append *k* elements having a weight of unity to the vector of uncertainties $\mathbf {s}$ of the pair-wise measurements to arrive at a new vector $\mathbf {s}^{\prime }$. We employ these appended matrices using the methods outlined in Sect. 3.4 to arrive at a new $(n+k)$-by-$(n+k)$ covariance matrix for the pair-wise data

35 $$\begin{aligned} \mathbf {\Sigma }^{\prime }_{d} = \mathbf {Q}^{\prime } \mathbf {\Sigma }^{\prime }_{\rho } \mathbf {Q}^{\prime \intercal } \end{aligned}$$

Next, we solve for the covariance matrix containing the relative uncertainties of the epoch-wise measurements by substituting $\mathbf {Q}^{\prime }$ and $\mathbf {\Sigma }^{\prime }_{d}$ for $\mathbf {Q}$ and $\mathbf {\Sigma }_{d}$, respectively, in Eq. (33):

36 $$\begin{aligned} \mathbf {\Sigma }^{\prime }_{\rho }&= {(\mathbf {Q}^{\prime \intercal } \mathbf {Q}^{\prime })}^{-1} \mathbf {Q}^{\prime \intercal } \mathbf {\Sigma }^{\prime }_{d} \mathbf {Q}^{\prime } {(\mathbf {Q}^{\prime \intercal } \mathbf {Q}^{\prime })}^{-1} \end{aligned}$$

The diagonal elements of this matrix lead to a vector of relative uncertainties of the epoch-wise measurements

37 $$\begin{aligned} \varvec{\sigma }_{\rho } = \text {diag}{(\mathbf {\Sigma }^{\prime }_{\rho })}^{1/2} \end{aligned}$$

We can also represent the corresponding correlation matrix of the epoch-wise measurements as

38 $$\begin{aligned} \mathbf {L}_{\rho } = \mathbf {D}_{\rho }^{-1/2} \mathbf {\Sigma }^{\prime }_{\rho } \mathbf {D}_{\rho }^{-1/2}, \end{aligned}$$

where $\mathbf {D}_{\rho }$ is the $(n+k)$-by-$(n+k)$ vertex-degree matrix defined similarly to its counterpart, the edge-degree matrix $\mathbf {D}$, as discussed in Sect. 3.4.

We continue with our simple example case of five epochs $\{t_{1},t_{2},t_{3},t_{4},t_{5}\}$ and $n=3$ pairs $\{e_{1,2},e_{3,4},e_{4,5}\}$ in $k=2$ distinct components, such that epochs $t_1$ and $t_2$ are vertices in the first component and epochs $t_3, t_4$, and $t_5$ are vertices in the second component (see Fig. 1g). We start by defining our constraint matrix $\mathbf {C}$ from Eq. (34):

39 $$\begin{aligned} \mathbf {C} = \begin{bmatrix} \frac{1}{2}&\quad \frac{1}{2}&\quad 0&\quad 0&\quad 0 \\ 0&\quad 0&\quad \frac{1}{3}&\quad \frac{1}{3}&\quad \frac{1}{3} \end{bmatrix} \end{aligned}$$

where $\mathbf {C}$ has 2 rows corresponding to the number of trees and the denominators of the fractional elements correspond to the number of epochs in each component, such that $\eta _1=2$ and $\eta _2=3$. We now can write the new system of equations as $\mathbf {Q}^{\prime } \mathbf {m} = \mathbf {d}^{\prime }$.

We also define a matrix $\mathbf {S}^{\prime }$ to include the uncertainty of the pair-wise data and the constraints:

40 $$\begin{aligned} \mathbf {S}^{\prime } = \begin{bmatrix} s_{1,2}&\quad 0&\quad 0&\quad 0&\quad 0 \\ 0&\quad s_{3,4}&\quad 0&\quad 0&\quad 0 \\ 0&\quad 0&\quad s_{4,5}&\quad 0&\quad 0 \\ 0&\quad 0&\quad 0&\quad 1&\quad 0 \\ 0&\quad 0&\quad 0&\quad 0&\quad 1 \end{bmatrix} \end{aligned}$$

Using Eq. (24), we modify the covariance matrix of pair-wise data $\mathbf {\Sigma }^{\prime }_{d}$:

41 $$\begin{aligned} \mathbf {\Sigma }^{\prime }_{{d}} = \begin{bmatrix} {s}_{1,2}^{2}&\quad 0&\quad 0&\quad 0&\quad 0 \\ 0&\quad {s}_{3,4}^{2}&\quad -\frac{{s}_{3,4}{s}_{4,5}}{2}&\quad 0&\quad 0 \\ 0&\quad -\frac{{s}_{3,4}{s}_{4,5}}{2}&\quad {s}_{4,5}^{2}&\quad 0&\quad 0 \\ 0&\quad 0&\quad 0&\quad \frac{1}{2}&\quad 0 \\ 0&\quad 0&\quad 0&\quad 0&\quad \frac{1}{3} \end{bmatrix} \end{aligned}$$

Defining an example set of pair-wise measurement uncertainties such that $s_{1,2} = s_{3,4} = s_{4, 5} = 1\text { (mm)}$, we can use Eq. (33) along with the substitutions in Eqs. (34) and (35) to arrive at the covariance matrix of relative epoch-wise measurements:

42 $$\begin{aligned} \mathbf {\Sigma }_{\rho } = \begin{bmatrix} \frac{3}{4}&\quad \frac{1}{4}&\quad 0&\quad 0&\quad 0 \\ \frac{1}{4}&\quad \frac{3}{4}&\quad 0&\quad 0&\quad 0 \\ 0&\quad 0&\quad \frac{2}{3}&\quad \frac{1}{6}&\quad \frac{1}{6} \\ 0&\quad 0&\quad \frac{1}{6}&\quad \frac{2}{3}&\quad \frac{1}{6} \\ 0&\quad 0&\quad \frac{1}{6}&\quad \frac{1}{6}&\quad \frac{2}{3} \end{bmatrix}(\text {mm}). \end{aligned}$$

### Least-squares solution using the pseudoinverse and normal equations

Having defined the data covariance matrix, the design matrix, and the model parameters, we can represent the weighted least-squares problem as minimizing the objective function

43 $$\begin{aligned} f_{obj}(\mathbf {d}; \mathbf {m}) = (\mathbf {Gm}-\mathbf {d})^{\intercal }\, \mathbf {\Sigma }_{d}^{-1} (\mathbf {Gm} - \mathbf {d}) \end{aligned}$$

To solve the weighted least-squares problem, we use the pseudoinverse:

44 $$\begin{aligned} \widetilde{\mathbf {m}} = {(\mathbf {G}^{\intercal } \mathbf {\Sigma }^{\dag }_{d} \mathbf {G})}^{\dag } \mathbf {G}^{\intercal } \mathbf {\Sigma }^{\dag }_{d} \mathbf {d} \end{aligned}$$

Since the two pseudoinverses always exist, the solution expressed in Eq. (44) determines a unique set of estimates for the model parameters.

To quantify the misfit, we calculate the mean squared error (MSE) of the fit, or variance of unit weight, from the scatter of the weighted residuals as Strang and Borre (1997):

45 $$\begin{aligned} \mathbf {\sigma }_0^2 = \frac{\mathbf {r}^{\intercal } \mathbf {\Sigma }^{\dag }_{d} \mathbf {r}}{n_G - m_G} , \end{aligned}$$

where the vector $\mathbf {r} = \mathbf {d} - \mathbf {G \widetilde{m}}$ refers to the residuals, and $n_G$ and $m_G$ refer to the number of rows and columns of $\mathbf {G}$, respectively, and $\nu = n_G - m_G$ refers to the degrees of freedom of the system.

The MSE is also called the reduced chi-squared statistic $\chi ^2$ (e.g., Aster et al. 2013). The scaled variance of the model parameters is

46 $$\begin{aligned} \mathbf {\Sigma }_{m} = \sigma _0^2 {(\mathbf {G}^{\intercal } \mathbf {\Sigma }^{\dag }_{d} \mathbf {G})}^{\dag } \end{aligned}$$

The estimated standard deviation of the model parameters is thus

47 $$\begin{aligned} \varvec{\sigma }_{m} = \sqrt{\text {diag}(\mathbf {\Sigma }_{m})}. \end{aligned}$$

### Applying the forward model

After calculating the solution to the temporal adjustment inverse problem, we apply the corresponding forward model to calculate the modeled values of displacement $\mathbf {d}_\mathrm{mod}$. We then integrate the corresponding temporal function *f*(*t*) over time to calculate the accumulated modeled displacement at each epoch in each tree. These values can then be plotted as a function of time. The constant of integration is arbitrarily assumed to be zero, thus setting $f(t_1) = 0$.

## Application to Okmok volcano

Temporal adjustment also applies to parameters in a geophysical model, such as the increase in volume of a magma chamber beneath a volcano (e.g., Lu and Dzurisin 2014; Feigl et al. 2014; Le Mével et al. 2015a). As a practical example, we apply our method of temporal adjustment to InSAR data collected from Okmok volcano in Alaska.

### Background of Okmok volcano

Several studies have analyzed geodetic data at Okmok volcano to make inferences about the magmatic “plumbing” at depth. Most of these studies describe the geodetic data using a model of a spherical source attributed to Mogi (1958) in a half-space with uniform values of the elastic properties and described by Segall (2010). This approach shows fluctuations in the estimated rate of magma influx between the eruptions in 1997 and 2008 (e.g., Fournier et al. 2009; Biggs et al. 2010; Lu et al. 2010).

### Setting up the inverse problem

The data set analyzed by Lu et al. (2005) includes SAR images acquired between July 1, 1997 and September 9, 2003 by four satellite missions: ERS-1, ERS-2, Radarsat-1, and JERS-1.

**Fig. 3:**
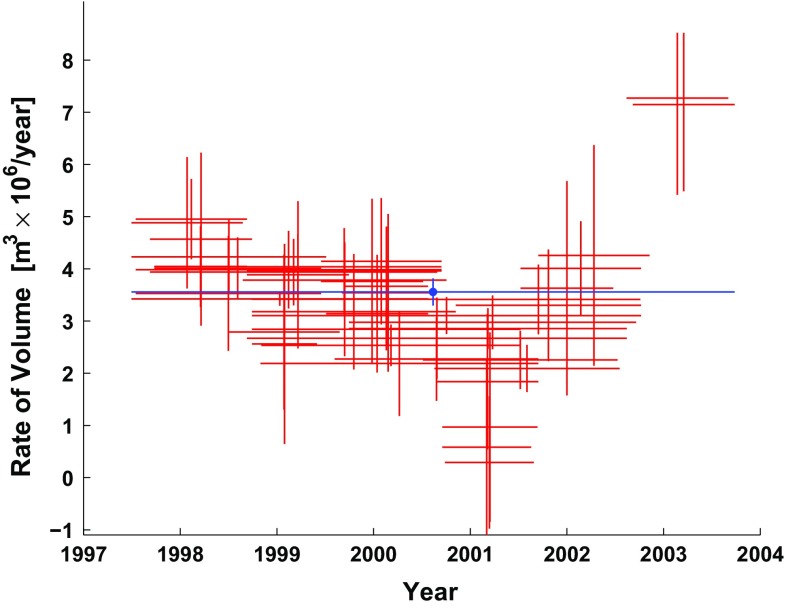
Rates of change in volume as a function of time for a modeled spherical magma chamber below Okmok volcano, as estimated from the 44 individual pairs of InSAR data (*red symbols*) and their weighted mean (*blue symbols*). *Horizontal segments* indicate observed time intervals. *Vertical bars* indicate the (unscaled) interval of 68 % confidence calculated from the RMSE values of the pair-wise solutions, as described in the text. Data from Table 1 of Lu et al. (2005) and reprinted with uncertainties in Online Resource 2

Although images acquired by ERS-1 form useful interferometric pairs with images acquired by ERS-2, other heterogeneous combinations do not form useful pairs. The data set of 45 epochs yields 44 interferometric pairs (Online Resource 2). For each of them, Lu et al. (2005) estimate the parameters in the Mogi model. We use these estimates of the volumetric rate of change to form our data set for inversion (Fig. 3). In other words, their output is our input. We convert their rates to differential volumes by multiplying each rate by its corresponding interval of time. This approach assumes that the time-dependent and position-dependent parts of the displacement field are separable (e.g., Feigl and Thurber 2009).

**Fig. 4:**
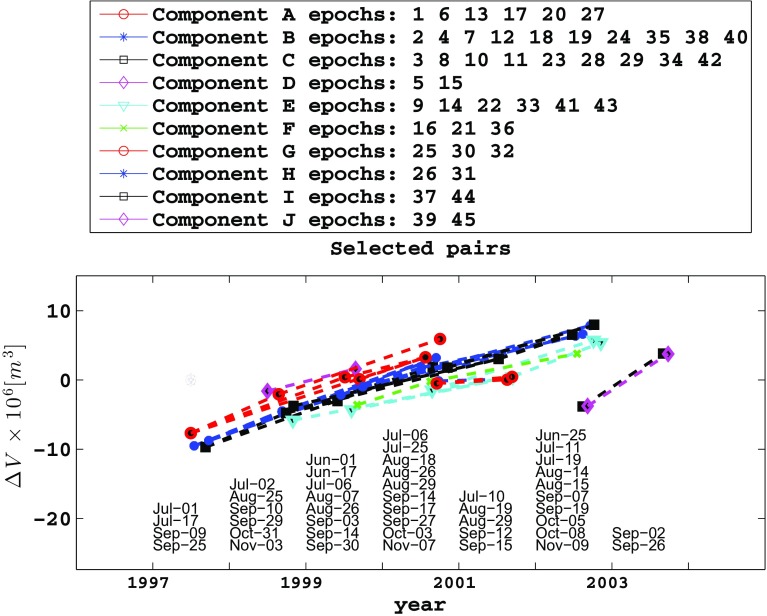
Plot of volume change for each pair as a function of time, showing epochs as *dots* and pairs as *line* segments connecting them. The calendar date for each epoch is listed in chronological order within each year

Lu et al. (2005) do not give uncertainties for their estimates. To set the a priori standard deviation of each datum, we weight each estimate in a relative sense. For each pair, we normalize the root mean squared error (RMSE) of the residuals (as given by Lu et al. (2005)) by the mean of the RMSE values averaged over all 44 pairs and multiplied by an arbitrary, constant scale factor of $\sim$ $10^6 \mathrm{m^3/year}$.

The graph of the data set includes $k=10$ distinct components. Figure 4 shows the volume change for each pair as a function of time, as calculated by findtrees.m and plotted by plottrees.m, as described in Online Resource 3.

Graphing the data groups them naturally into subsets according to the sensors. For example, pairs of SAR images acquired by the ERS-2 satellite mission fall into components A through F, whereas images acquired by the RADARSAT-1 satellite fall into components G though J. Images are further separated into components by tracks. In estimating the model parameters, Lu et al. (2005) account for the different imaging configurations (e.g., incidence angle, radar wavelength) for each pair individually.

### Estimation of epoch-wise variance

**Fig. 5:**
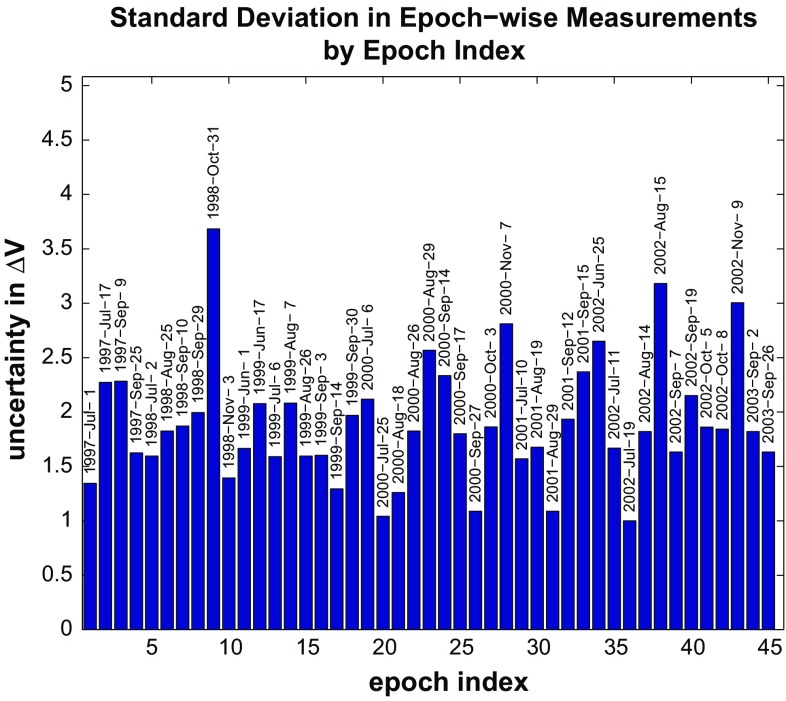
*Bar graph* of relative standard deviations in epoch-wise measurements as determined from the formulation in Sect. 3.6. The relative standard deviations are normalized by the smallest deviation. Each *bar* is labeled with the corresponding calendar date of the epoch

Using Eq. (36), we calculate the relative covariance matrix of the epoch-wise measurements. By taking the square root of the diagonal elements of this covariance matrix, we are able to determine the uncertainty of each of the individual, epoch-wise measurements. A plot of these values is shown in Fig. 5. The largest relative uncertainties occur at epochs during the winter season, during which snow on the ground, precipitation, and/or moisture in the atmosphere are common in Alaska. These effects tend to degrade the quality of the interferogram, and thus the overall misfit of the modeled phase values to the observed values, found by Lu et al. (2005).

### Single-segment secular rate parameterization

**Fig. 6:**
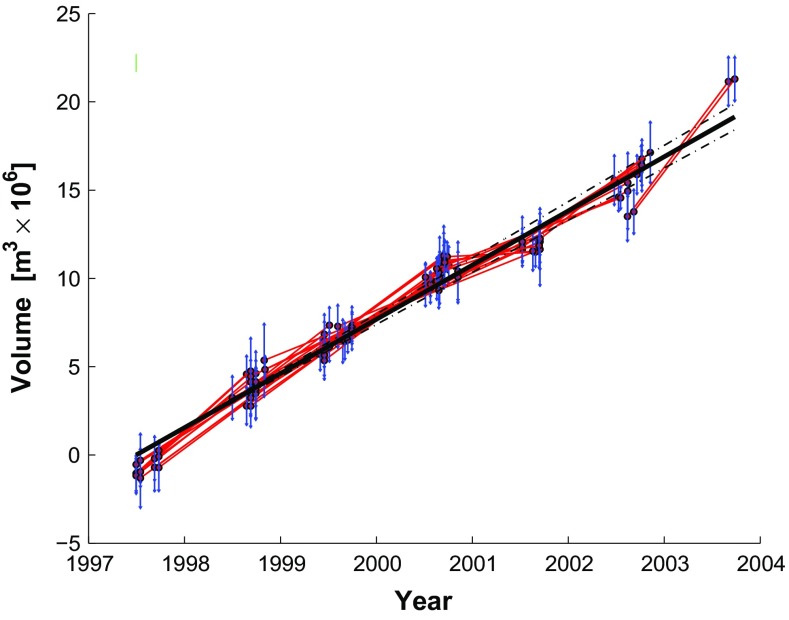
Volume increase as a function of time as estimated by temporal adjustment using a single-parameter model for a constant (secular) rate. *Black lines* show modeled value (*solid line*) with the envelope of 69 % confidence, after scaling by $\sigma _0$. The *green tick* represents the reference epoch. *Red segments* indicate the differential change in volume estimated from individual pairs. For each pair, the volume at the mid-point of the time interval is plotted to fall on the modeled curve. The estimated volume for each pair is plotted with its associated uncertainty (*vertical blue bars*). The length of each *blue bar* is set a posteriori to the 68 % confidence interval for the pair after scaling by $\sigma _0/\sqrt{2}$, where $\sigma _0 = 1.0226$

We consider six different parameterizations for the temporal adjustment. To save space, we show plots for only five of them in this paper. Plots for all the examples appear in the documentation for the GraphTreeTA software that is available at GitHub. The simplest parameterization is a constant-rate (secular) parameterization with a single-element parameter vector $\mathbf {m}$. Following the method outlined in Sect. 3.3, we construct the design matrix $\mathbf {G}$ with a temporal function where $t_0$ is the initial epoch. Using the $n=44$ pair-wise data, we find a good fit with misfit $\sigma _0 = 1.0226$. Results are shown in Fig. 6.

### Five-segment piecewise-linear parameterization

We expand the linear parameterization by adding 5 segments delimited by a 6-element vector of break points $\mathbf {t}_{b}$ containing a break on January 1st of each year from 1999 to 2002, inclusive. The temporal function becomes piecewise-linear:

48 $$\begin{aligned} m_j f_{5\mathrm{seg}}^{(j)}(t_i) = m_j {\left\{ \begin{array}{ll} (t_i-t_{b_j}) &{} \text {if } t_{b_j} \le t_i < t_{b_{j+1}} \\ (t_{b_{j+1}}-t_{b_j}) &{} \text {if }t_i \ge t_{b_{j+1}} \\ 0 &{} \text {otherwise} \end{array}\right. } \end{aligned}$$

**Fig. 7:**
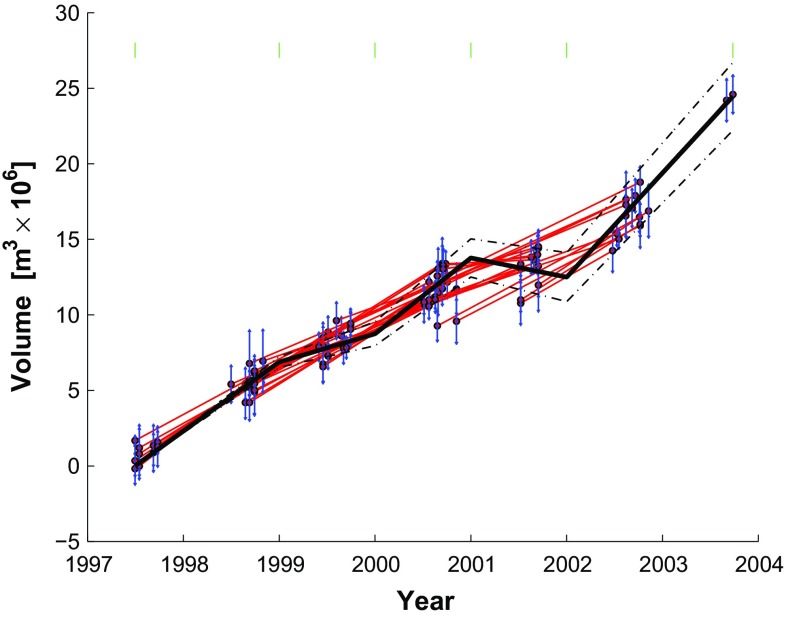
Volume increase as a function of time as estimated by temporal adjustment using the 5-segment piecewise-linear parametrization with yearly breaks from January 1, 1999 to January 1, 2002. The *green ticks* represent epochs separating time intervals. Misfit $\sigma _0=0.4034$. This figure corresponds with Figure 6.98 of Lu and Dzurisin (2014). Plotting conventions as in previous figure

This expression defines a 5-element parameter vector $\mathbf {m}$, where $m_j$ describes the rate for the $j{\text {th}}$ interval, and a design matrix $\mathbf {G}$ with 5 columns. The element $G_{i,j}$ stores the value of $f_{5\mathrm{seg}}(t_i)$ evaluated for the $i{\text {th}}$ epoch and the $j{\text {th}}$ segment. This solution includes 5 rates and a misfit of $\sigma _0 = 0.4034$, as seen in Fig. 7. This figure essentially reproduces the result of Lu and Dzurisin (2014) (their Figure 6.98).

We find a better fit using 5 segments than using the constant (secular) rate parameterization. To decide if the additional complexity is justified, we perform an F test (e.g., Wackerly et al. 2007). The null hypothesis states that the two sets of weighted residuals (the observed minus calculated values of the differential volumes normalized by their corresponding measurement uncertainties) have equal variance. Comparing the secular rate and 5-segment models, we find $F = 36.07$. Since the critical value of the F statistic for a significance level of $\alpha = 0.05$ and degrees of freedom $\nu _1 = n - 1 = 43$ and $\nu _2 = n - 5 = 39$ (where *n* is the number of calculated residuals) is $F_{\alpha , \nu _1, \nu _2} = 1.69$, the null hypothesis is rejected with 95 % confidence. We conclude that the 5-segment model provides an appropriate level of complexity.

In addition, we perform a two-tailed Student’s t test to decide whether or not the rates estimated during successive intervals of the 5-segment model show significant differences (e.g., Wackerly et al. 2007). The null hypothesis states that the mean rates during the successive year-long intervals before and after January 1st of the tested year are equal. We set the significance level at $\alpha = 0.05$. We find that the value of the test statistic lies outside the acceptance interval $[-T_{\alpha /2}, +T_{\alpha /2}]$ formed by the corresponding values of $\pm T_{\alpha /2}$ for each year. Thus, the five estimated rates are distinguishable.

### Berardino parameterization

**Fig. 8:**
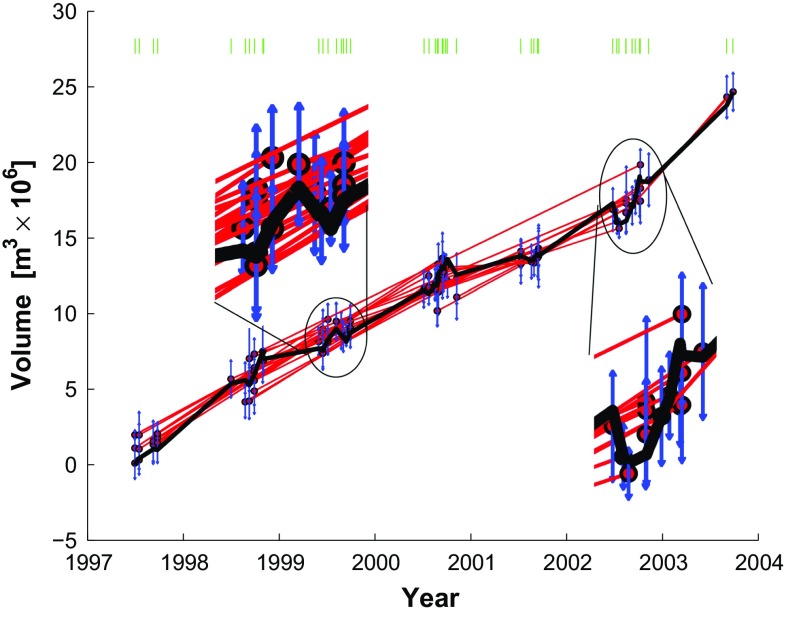
Volume increase as a function of time as estimated by temporal adjustment using the method of rates parameterized in terms of 44 velocity parameters as developed by Berardino et al. (2002). Enlarged portions show the local oscillatory nature of the SVD minimum-length solution. Plotting conventions as in previous figure

We apply the parameterization in terms of rate (Berardino et al. 2002), as outlined in Sect. 3.2. With this parameterization, the number of parameters is greater than the number of data such that $m_G > n_G$. Consequently, SVD yields a locally oscillatory solution (Fig. 8). For the same reason, the variance of unit weight $\sigma ^2$ cannot be calculated from Eq. (45). Instead, we interpret the null residuals as a perfect fit and set the misfit $\sigma _0 = 0$.

**Fig. 9:**
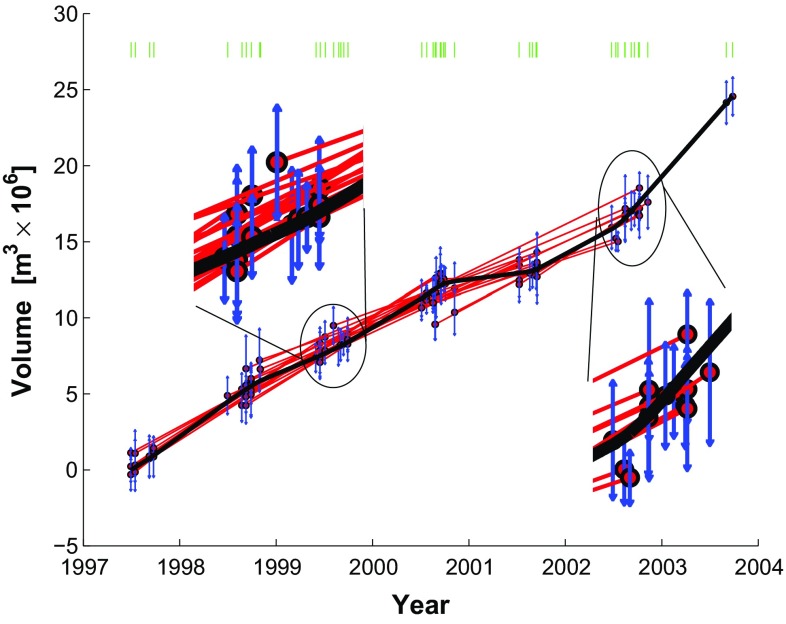
Volume increase as a function of time as estimated by temporal adjustment using the method of rates parameterized in terms of 44 velocity parameters as developed by Berardino et al. (2002) with first-order Tikhonov regularization to flatten the solution. We choose the regularization parameter $\beta =0.0010$ based on an L-curve plot of the norm of the residuals against the norm of the parameter vector (e.g., Aster et al. 2013). Plotting conventions as in previous figure

In addition, we apply first-order Tikhonov regularization to favor a constant-rate solution found using the method of Berardino et al. (2002) (Fig. 9). We experiment with several values of smoothing parameters to find the solution to the weighted least-squares problem shown in Fig. 9. We choose the regularization parameter $\beta = 0.0010$ based on the L-shaped curve of the norm of the residuals plotted as a function of the norm of the parameter vector to balance the trade-off between fitting the data and reducing the roughness. Comparing the enlarged sections of Figs. 8 and 9, we see that the Tikhonov regularization mitigates the artifactual oscillations during the summers between 1999 and 2001. The sharp increase in rate beginning in summer 2001 remains apparent in Fig. 9.

### Exponential decay parameterization

We parameterize the time dependence as an exponentially decaying rate via Eq. (21). This formulation is compatible with viscoelastic relaxation, as suggested by previous studies of Okmok volcano (e.g., Fournier et al. 2009; Masterlark et al. 2010, 2016). We set $t_0$ at May 23, 1997, the end of the 1997 eruption. To estimate the best-fitting characteristic time scale $\tau _m\mathop {=}\limits ^{.} 6$ years, we use nonlinear optimization via an “interior point” algorithm (e.g., Byrd et al. 2000) implemented in MATLAB (2014). This exponential model produces a slightly better fit ($\sigma _0 = 0.9455$, Online Resource 4) than the single-segment (constant-rate) model.

### Modified exponential parameterization

**Fig. 10:**
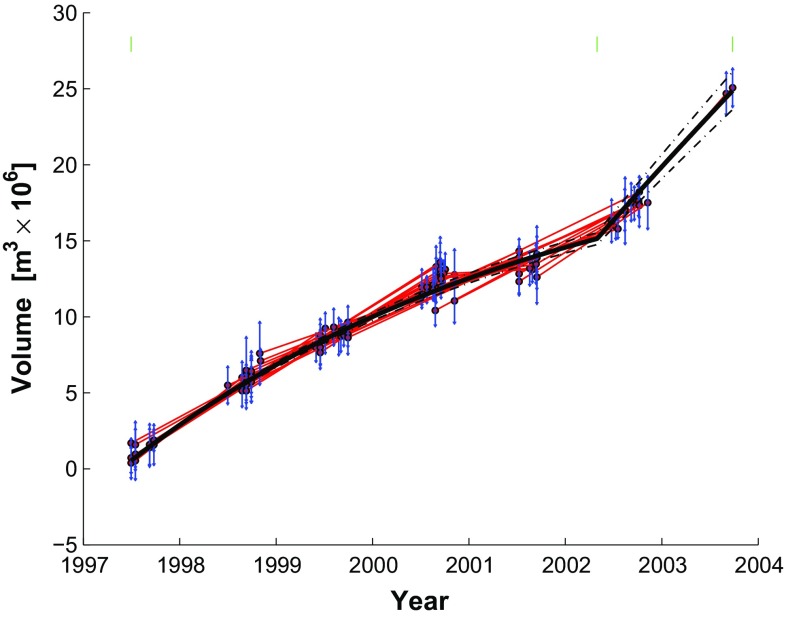
Volume increase as a function of time as estimated by temporal adjustment using the parametrization in terms of an exponentially decaying rate until June 21, 2002 and then a constant secular rate through the end of the data set at September 26, 2003. The characteristic time scale is $\tau _m = 4.9$ years. Misfit $\sigma _0 = 0.5685$. Plotting conventions as in previous figure

In the GPS time series, Fournier et al. (2009) observe a rapid pulse of inflation beginning in the summer of 2002 (specifically stations OKCD and OKCE) (their Figure 6) which is not consistent with the previous exponential trend. To account for it, we modify the exponential parameterization by adding a secular rate for the interval from June 21, 2002 to September 26, 2003, the last epoch in the time series. We define this interval by letting $t = t_{s1}$ and $t = t_{s2}$, corresponding to the beginning and end of the time span, respectively. The results of nonlinear optimization for an exponentially decaying rate until June 21, 2002 indicate a new best-fitting characteristic time scale of $\tau _{m} \mathop {=}\limits ^{.} 5$ years. Extending Eq. (21), we choose a temporal function such that

49 $$\begin{aligned} \mathbf {m}f_{m\mathrm{exp}}(t_i) = {\left\{ \begin{array}{ll} m_1 \left( 1 - \text {exp}(-\frac{t_i - t_0}{\tau _{m}})\right) &{} \quad \text {if } t_i < t_{s1} \\ m_2(t_i - t_{s1}) &{} \quad \text {if } t_{s1} \le t_i \le t_{s2} \\ 0 &{}\quad \text {otherwise} \end{array}\right. } \end{aligned}$$

where the design matrix $\mathbf {G}$ has three columns corresponding to the three elements in the parameter vector $\mathbf {m}$. This modification improves the fit to $\sigma _0 = 0.5685$ (Fig. 10).

To determine if the modification is justified, we perform an F test on the exponential decay and the modified exponential model (e.g., Wackerly et al. 2007). The null hypothesis states that the two sets of weighted residual values of displacement have equal variance. We find $F = 13.67$. Since the critical value of the F statistic for a significance level of $\alpha = 0.05$ and degrees of freedom $\nu _1 = n - 1 =43$ and $\nu _2 = n - 3 =41$ is $F_{\alpha , \nu _1, \nu _2} = 1.67$, the null hypothesis is rejected with 95 % confidence. We conclude that the additional complexity of the modified exponential model is justified.

Next, we compare the modified exponential model with the 5-segment piecewise-linear parameterization. We know that the misfit of the 5-segment piecewise-linear model is less than that of the modified exponential decay, but we must also consider their variances. We test the null hypothesis that the two sets of weighted residual values of displacement have equal variance. With a significance level of $\alpha = 0.05$ and degrees of freedom $\nu _1=42$ and $\nu =39$, the critical value of the F statistic is $F_{\alpha , \nu _1, \nu _2} = 1.69$. Since the calculated F value is 4.0211, and the null hypothesis is rejected with 95 % confidence. Thus, we conclude that the 5-segment piecewise-linear parameterization provides a significantly better fit.

## Discussion

Among the various parameterizations for Okmok, the empirical 5-segment piecewise linear model provides the best fit. The second-best fit is a modified exponential function, and suggests viscoelastic relaxation following the 1997 eruption, with an intrusion starting mid-2002 and ending in late 2003 (e.g., Jellinek and DePaolo 2003).

Between June of 2002 and September 2003, the estimated rate of volumetric increase is $6.2\,\pm \,0.6\times 10^6\mathrm{m^3/year}$. This result is consistent with the suggestion of a “pulse of rapid inflation” from “summer 2002 to late 2003” (Fournier et al. 2009; Biggs et al. 2010). If viscoelastic relaxation also occurred in the years following this pulse, then we would expect slower inflation and/or deflation in later years (e.g., Masterlark et al. 2016). In this case, the characteristic time scale would be of the same order of magnitude as the ratio of Maxwell viscosity to rigidity (e.g., Hetland and Hager 2005). Alternatively, Fournier (2008) suggests degassing to explain the slowing rate of inflation that began in 2004. Another possibility is that the viscosity of magma flowing upward through a conduit into a shallow reservoir determines the characteristic time scale (e.g., Le Mével et al. 2015b).

## Conclusions

We have shown that graph theory is useful for analyzing pair-wise InSAR data in the temporal domain. In particular, the normalized edge Laplacian matrix calculated from the edge-vertex incidence matrix of the graph of the data set represents its correlation.

This formulation also leads to the covariance matrix of the epoch-wise measurements to calculate their relative uncertainties. For example, the Okmok data set shows greater uncertainty for single-epoch, individual SAR images acquired during the winter season than for those in the summer. Although mathematically straightforward, this derivation has not been previously applied to InSAR data.

If the number of distinct trees or components is greater than one, then a piecewise linear parameterization in terms of rates, as proposed by Berardino et al. (2002), leads to a locally oscillatory solution. To mitigate this issue, we use first-order Tikhonov regularization.

Using graph theory, we have derived a result for the pair-wise data covariance matrix which agrees with previous formulae while providing useful insight into the graphical structure of the data. Moreover, the formulation is concise and independent of the choice of model.

The formulation in terms of incidence graphs also applies to any quantity derived from pair-wise differences. For example, plots of orbital separation $\text {B}_{\perp }$ as a function of time are calculated with respect to a “virtual” reference orbit with a constraint of zero mean, as shown in Fig. 4 (e.g., Fialko et al. 2002). Similarly, one could apply temporal adjustment to individual, co-located pixels in a time series of interferograms or even their decomposition into wavelets (e.g., Jolivet et al. 2015).

## Electronic supplementary material

Below is the link to the electronic supplementary material.

Supplementary material 1 (pdf 235 KB)

Supplementary material 2 (pdf 183 KB)

Supplementary material 3 (pdf 89 KB)

Supplementary material 4 (pdf 146 KB)
