# Supplementary material for: Graph theory for analyzing pair-wise data: application to geophysical model parameters estimated from interferometric synthetic aperture radar data at Okmok volcano, Alaska
Source: J Geod. 2016 Jul 9;91(1):9–24. doi: 10.1007/s00190-016-0934-5 (PMC7045901; doi:10.1007/s00190-016-0934-5)
Supplement: Supplementary file 1 — Supplementary material 1 (pdf 235 KB) [file 190_2016_934_MOESM1_ESM.pdf]

**Online Resource 1.** Table of mathematical symbols.

| Symbol | Meaning |
|---|---|
| $\mathbf{A}^{\dagger}$ | pseudoinverse of $\mathbf{A}$ |
| $\mathbf{A}^{T}$ | transpose of $\mathbf{A}$ |
| $\mathbf{A}^{-1}$ | inverse of $\mathbf{A}$ |
| $\mathbf{B}$ | pair-rate incidence matrix |
| $B_{\perp}$ | orbital separation, perpendicular baseline |
| $\mathbf{C}$ | constraint matrix with $k$ rows |
| $c$ | number of pair-wise combinations as defined in Feigl and Thurber (2009) |
| $corr$ | correlation coefficient |
| $\mathbf{D}$ | $n$-by-$n$ diagonal matrix of degrees in graph of data set |
| $\mathbf{d}$ | $n$-by-1 data vector |
| $\mathbf{d_{con}}$ | $k$-by-1 vector of data constraints |
| $\mathbf{d_{mod}}$ | vector of modeled displacement |
| $\mathbf{d_{obs}}$ | $n$-by-1 vector of observed data |
| $diag$ | operator transforming a vector into a diagonal matrix |
| $e_i$ | $i^{\text{th}}$ element (or pair) of $E$ |
| $F_{\alpha,\nu_1,\nu_2}$ | critical value for F test |
| $f_{obj}(\mathbf{d};\mathbf{m})$ | objective function for weighted least-squares |
| $f(t)$ | temporal function |
| $\mathbf{G}$ | $n$-by-$m$ design matrix |
| $\mathbf{G}^{[\text{B}]}_{n\times(m-1)}$ | Berardino et al. (2002) design matrix |
| $\mathbf{K}$ | $n$-by-$n$ edge-version of the Laplacian |
| $k$ | number of components of a disconnected graph |
| $\mathbf{L}$ | normalized edge-version of the Laplacian |
| $\mathbf{L}_{\rho}$ | correlation matrix of epoch-wise measurements |
| $\mathbf{m}$ | $m$-by-1 vector of model parameters |
| $m$ | number of elements in parameter vector |
| $\widetilde{\mathbf{m}}$ | estimated model parameter vector |
| $m_G$ | number of columns of $\mathbf{G}$ |
| $n$ | number of elements in data vector |
| $n_G$ | number of rows of $\mathbf{G}$ |
| $n$-by-$m$ | matrix having $n$ rows and $m$ columns |
| $\mathbf{Q}$ | $n$-by-$q$ edge-vertex incidence matrix |
| $q$ | number of epochs as defined in Feigl and Thurber (2009) |
| $q_{i,j}$ | element in the $i^{\text{th}}$ row and $j^{\text{th}}$ column of $\mathbf{Q}$ |
| $\mathbf{r}$ | vector of residuals |
| $rank(\mathbf{Q})$ | rank of the incidence matrix |
| RMSE | root-mean-squared error |
| $\mathbf{S}$ | $n$-by-$n$ diagonal matrix of sample standard deviation of pair-wise measurement errors |
| SVD | singular value decomposition |
| $\mathbf{T}$ | $m$-by-$m$ diagonal matrix of time intervals |
| $\mathbf{t}$ | vector of unique epochs in chronological order |
| $t_0$ | reference epoch for exponentially decaying rate parameterization |
| $t_i$ | the $i^{\text{th}}$ epoch, or the $i^{\text{th}}$ element of $\mathbf{t}$ |
| $t_q$ | predefined reference epoch |
| $t_{s1}$ | lower bound epoch for modified exponentially decaying rate parameterization |

| | |
|---|---|
| $t_{s2}$ | upper bound epoch for modified exponentially decaying rate parameterization |
| $T_{\nu,\frac{\alpha}{2}}$ | critical value for two-tailed Student t-test |
| $\mathbf{v}$ | vector/set of rate parameters having $(m-1)$ elements |
| $v_i$ | $i^{\text{th}}$ element (or vertex) of $V$ |
| $\mathbf{W}$ | matrix quantifying the solution roughness in Tikhonov regularization |
| $\alpha$ | significance level |
| $\beta$ | Tikhonov regularization parameter |
| $\boldsymbol{\Delta}$ | edge-vertex matrix of path |
| $\delta_{i,j}$ | the $(i,j)^{\text{th}}$ element of the Kronecker delta |
| $\eta_i$ | number of epochs in the $i^{\text{th}}$ component |
| $\mu$ | rank deficiency of an underdetermined system |
| $\nu$ | degrees of freedom |
| $\nu_1$ | numerator degrees of freedom (F test) |
| $\nu_2$ | denominator degrees of freedom (F test) |
| $\rho$ | vector of range |
| $\rho(t_i)$ | range at epoch $t_i$ |
| $\boldsymbol{\Sigma}_{\mathbf{d}}$ | covariance matrix of pair-wise data |
| $\boldsymbol{\Sigma}_{\mathbf{m}}$ | scaled covariance matrix of model parameters |
| $\sigma_{\mathbf{m}}$ | estimated standard deviation of model parameters |
| $\sigma_\rho$ | vector of relative uncertainties of epoch-wise measurements |
| $\boldsymbol{\Sigma}'_\rho$ | covariance matrix of relative epoch-wise measurements |
| $\sigma_0^2$ | fit, or variance of unit weight, as calculated from weighted residual scatter |
| $\sigma_r^2$ | mean of the RMSEs of the Okmok data set |
| $\tau_m$ | characteristic time constant for exponential parameterizations |
| $\chi^2$ | chi-squared test statistic |

List of alphabetically sorted mathematical symbols used throughout the text with English alphabet letters appearing before Greek alphabet letters.

## References


Berardino, P., G. Fornaro, R. Lanari, and E. Sansosti (2002), A new algorithm for surface deformation monitoring based on small baseline differential SAR interferograms, *Geoscience and Remote Sensing, IEEE Transactions on*, *40*(11), 2375–2383, DOI 10.1109/TGRS.2002.803792

Feigl, K. L., and C. H. Thurber (2009), A method for modelling radar interferograms without phase unwrapping: application to the M 5 Fawnskin, California earthquake of 1992 December 4, *Geophysical Journal International*, *176*(2), 491–504, DOI 10.1111/j.1365-246X.2008.03881.x
