# Supplementary material for: Graph theory for analyzing pair-wise data: application to geophysical model parameters estimated from interferometric synthetic aperture radar data at Okmok volcano, Alaska
Source: J Geod. 2016 Jul 9;91(1):9–24. doi: 10.1007/s00190-016-0934-5 (PMC7045901; doi:10.1007/s00190-016-0934-5)
Supplement: Supplementary file 2 — Supplementary material 2 (pdf 183 KB) [file 190_2016_934_MOESM2_ESM.pdf]

Online Resource 2. Okmok dataset from Lu et al. (2005) with a priori uncertainties of pair-wise measurements.

ID	Orbit	Year	Month	Day	Orbit	Year	Month	Day	Track	Bn_in_m	dV_m^3	RMSE, mm	dV/dt (m^3)/yr	σ (m^3)/yr
1	106773	1992	10	31	112284	1993	11	20	115	13	7.65E+06	12.7	7.26E+06	2.42E+06
4	110738	1993	8	4	121603	1995	9	1	72	-154	3.55E+06	9.7	1.71E+06	9.39E+05
5	110738	1993	8	4	207441	1996	9	27	72	-136	2.46E+06	8.2	7.81E+05	5.23E+05
6	110781	1993	8	7	122147	1995	10	9	115	304	3.71E+06	8	1.71E+06	7.40E+05
7	111239	1993	9	8	122104	1995	10	6	72	-230	2.99E+06	9.2	1.44E+06	8.90E+05
8	111282	1993	9	11	122147	1995	10	9	115	-97	2.97E+06	10	1.43E+06	9.68E+05
9	111740	1993	10	13	122104	1995	10	6	72	-24	2.33E+06	8.2	1.18E+06	8.32E+05
10	111740	1993	10	13	207441	1996	9	27	72	-339	2.32E+06	10.1	7.85E+05	6.87E+05
11	111783	1993	10	16	122147	1995	10	9	115	-72	2.08E+06	7.8	1.05E+06	7.92E+05
12	112012	1993	11	1	122376	1995	10	25	344	71	1.95E+06	5.4	9.84E+05	5.48E+05
13	112012	1993	11	1	202703	1995	10	26	344	154	1.65E+06	6	8.32E+05	6.08E+05
14	121603	1995	9	1	207441	1996	9	27	72	18	-1.34E+06	4.5	-1.25E+06	8.44E+05
21	210719	1997	5	8	217733	1998	9	10	344	167	7.11E+06	5.1	5.30E+06	7.64E+05
22	211492	1997	7	1	217504	1998	8	25	115	236	5.62E+06	7.2	4.88E+06	1.26E+06
23	211492	1997	7	1	222013	1999	7	6	115	-119	8.52E+06	7.1	4.23E+06	7.09E+05
24	211492	1997	7	1	227524	2000	7	25	115	-72	1.05E+07	2	3.42E+06	1.31E+05
25	211721	1997	7	17	217733	1998	9	10	344	69	5.70E+06	4.4	4.95E+06	7.69E+05
26	211721	1997	7	17	221741	1999	6	17	344	48	6.77E+06	10.5	3.53E+06	1.10E+06
27	211721	1997	7	17	228254	2000	9	14	344	-41	1.26E+07	11.7	3.98E+06	7.44E+05
28	212494	1997	9	9	218005	1998	9	29	115	190	4.82E+06	8.7	4.57E+06	1.66E+06
29	212494	1997	9	9	228025	2000	8	29	115	-80	1.17E+07	9.4	3.94E+06	6.36E+05
30	212723	1997	9	25	217733	1998	9	10	344	92	3.88E+06	3.7	4.05E+06	7.76E+05
31	212723	1997	9	25	221741	1999	6	17	344	-25	6.92E+06	5.1	4.01E+06	5.94E+05
32	212723	1997	9	25	228254	2000	9	14	344	-18	1.20E+07	9.2	4.04E+06	6.22E+05
33	216731	1998	7	2	222743	1999	8	26	344	-251	3.21E+06	8.5	2.79E+06	1.48E+06
34	217504	1998	8	25	228526	2000	10	3	115	-11	7.98E+06	7.4	3.79E+06	7.06E+05
35	217733	1998	9	10	223244	1999	9	30	344	295	4.10E+06	7.4	3.89E+06	1.41E+06
36	217733	1998	9	10	228254	2000	9	14	344	-110	7.97E+06	8.2	3.96E+06	8.19E+05
37	217733	1998	9	10	238274	2002	8	15	344	216	1.05E+07	15.2	2.67E+06	7.78E+05
38	218005	1998	9	29	221512	1999	6	1	115	77	1.72E+06	6.4	2.56E+06	1.92E+06
39	218005	1998	9	29	228025	2000	8	29	115	-270	6.55E+06	10.4	3.42E+06	1.09E+06
40	218005	1998	9	29	229027	2000	11	7	115	-147	6.70E+06	11.6	3.18E+06	1.11E+06
41	218005	1998	9	29	232534	2001	7	10	115	120	7.90E+06	5.6	2.84E+06	4.05E+05
42	218005	1998	9	29	239047	2002	10	8	115	56	1.25E+07	7.1	3.11E+06	3.55E+05
43	218463	1998	10	31	233493	2001	9	15	72	-11	6.29E+06	14.4	2.19E+06	1.01E+06
44	218506	1998	11	3	232534	2001	7	10	115	-90	6.80E+06	5.3	2.54E+06	3.97E+05
46	221741	1999	6	17	227252	2000	7	6	344	-217	3.96E+06	8.3	3.76E+06	1.58E+06
47	221741	1999	6	17	228254	2000	9	14	344	7	5.16E+06	7.5	4.15E+06	1.21E+06
48	222013	1999	7	6	227524	2000	7	25	115	47	3.31E+06	5.9	3.14E+06	1.13E+06
49	222471	1999	8	7	233493	2001	9	15	72	-283	4.79E+06	8.4	2.27E+06	8.01E+05
50	222850	1999	9	3	227860	2000	8	18	451	108	3.39E+06	7.2	3.54E+06	1.51E+06
51	223015	1999	9	14	227524	2000	7	25	115	274	3.16E+06	4.9	3.67E+06	1.14E+06
52	223244	1999	9	30	238274	2002	8	15	344	-79	8.21E+06	5.6	2.86E+06	3.92E+05
53	223244	1999	9	30	238775	2002	9	19	344	33	8.84E+06	7.6	2.98E+06	5.14E+05
55	227252	2000	7	6	237773	2002	7	11	344	-187	4.54E+06	5.6	2.26E+06	5.59E+05
56	227982	2000	8	26	233493	2001	9	15	72	402	1.94E+06	6.8	1.84E+06	1.30E+06
57	227982	2000	8	26	239004	2002	10	5	72	75	7.20E+06	7	3.41E+06	6.67E+05
58	229027	2000	11	7	239047	2002	10	8	115	203	6.33E+06	10.2	3.30E+06	1.07E+06
59	232534	2001	7	10	237544	2002	6	25	115	-183	3.48E+06	9.8	3.63E+06	2.05E+06
60	232534	2001	7	10	239047	2002	10	8	115	-64	5.00E+06	5.6	4.01E+06	9.03E+05
61	233493	2001	9	15	239505	2002	11	9	72	93	4.90E+06	12.1	4.26E+06	2.11E+06
65	227860	2000	8	18	237880	2002	7	19	451	8	4.01E+06	4.3	2.09E+06	4.51E+05
66	122147	1995	10	9	212494	1997	9	9	115	8	-4.82E+07	9.8	-2.51E+07	1.03E+06
67	122147	1995	10	9	212494	1997	9	9	115	8	-4.73E+07	8.7	-2.47E+07	9.12E+05
68	325570	2000	9	27	330372	2001	8	29	800	-118	2.68E+05	5.8	2.91E+05	1.27E+06
70	325420	2000	9	17	330222	2001	8	19	900	362	5.39E+05	7.7	5.86E+05	1.68E+06
71	325420	2000	9	17	330565	2001	9	12	900	350	9.55E+05	8.9	9.69E+05	1.82E+06
74	403682	1992	10	13	435314	1998	7	26	777	240	-3.39E+07	10.2	-5.86E+06	3.55E+05

“Dates are image acquisition times. Orbit numbers include the satellite ID (1, ERS1; 2, ERS2; 3, RADARSAT1; and 4, JERS1) and orbit on which the images were acquired. The same track number applies to both images in an InSAR pair. B_n is the perpendicular component of the baseline with respect to the SAR look angle. dV is the volume change of the source of the best fitting model in the case that the model source in three dimensions was fixed. RMSE is the root-mean-square error between the observed and modeled interferograms. Interferograms 66 and 67 are from the same, original interferogram. Phase values in the original interferogram, however, are discontinuous between two major patches which are represented by interferograms 66 and 67, respectively. So, interferograms 66 and 67 are treated as two different images.” (*Lu et al.*, 2005). In our analysis, we abridge the dataset to include only pairs with acquisitions after the end of the 1997 eruption (May 25, 1997). The volume change rate per pair in column 14 is found by dividing the volumetric estimate by the corresponding time interval. The uncertainty of volume change rate in column 15 is estimated by scaling the individual RMSE values by their mean $\sigma_r = 4.98$ [mm].

References

Lu, Z., T. Masterlark, and D. Dzurisin (2005), Interferometric synthetic aperture radar study of Okmok volcano, Alaska, 1992–2003: Magma supply dynamics and postemplacement lava flow deformation, *Journal of Geophysical Research: Solid Earth*, *110*(B2), DOI 10.1029/2004JB003148
