# Supplementary material for: Graph theory for analyzing pair-wise data: application to geophysical model parameters estimated from interferometric synthetic aperture radar data at Okmok volcano, Alaska
Source: J Geod. 2016 Jul 9;91(1):9–24. doi: 10.1007/s00190-016-0934-5 (PMC7045901; doi:10.1007/s00190-016-0934-5)
Supplement: Supplementary file 3 — Supplementary material 3 (pdf 89 KB) [file 190_2016_934_MOESM3_ESM.pdf]

Online Resource 3: Implementation

We have implemented the method described in Section 3 using *MATLAB* (2014). Our source code is available free of charge and can be found on GitHub under the General Inversion of Phase Technique (GIPhT) suite (*https://github.com/feigl/gipht*). It is licensed under the GNU Lesser General Public License. The accompanying documentation includes figures showing all of the solutions discussed here (*https://uwmadison.app.box.com/files/0/f/4061526069/GIPhTBox*).

Given the edge Laplacian matrix $\mathbf{L}$ of the data, the function *incidence_to_cov.m* calculates the data covariance matrix according to equations (22) through (24) of Section 3.4.

The function *pinveb.m* computes the pseudoinverse of a matrix using singular value decomposition and returns its condition number. An input argument specifies a threshold for truncating the singular values; otherwise a default tolerance is used. Alternatively, one can specify the number of singular values to include in the solution. This function also plots the spectrum of singular values.

The function *ls_with_cov.m* computes the solution to the weighted least-squares problem using the pseudoinverse via equations (44) through (47) in Section 3.7.

The function *findtrees.m* finds and counts the distinct trees of a data set using the rank deficiency μ of the system and the rational basis of the null space of $\mathbf{Q}$.

The function *plottrees.m* plots the graph of the data set as distinct trees (e.g., Figure 4).

References

MATLAB (2014), version 8.3.0.532 (R2014a), *The MathWorks Inc.*, Natick, Massachusetts. URL http: //www.mathworks.com/products/matlab
