# Supplementary material for: Graph theory for analyzing pair-wise data: application to geophysical model parameters estimated from interferometric synthetic aperture radar data at Okmok volcano, Alaska
Source: J Geod. 2016 Jul 9;91(1):9–24. doi: 10.1007/s00190-016-0934-5 (PMC7045901; doi:10.1007/s00190-016-0934-5)
Supplement: Supplementary file 4 — Supplementary material 4 (pdf 146 KB) [file 190_2016_934_MOESM4_ESM.pdf]

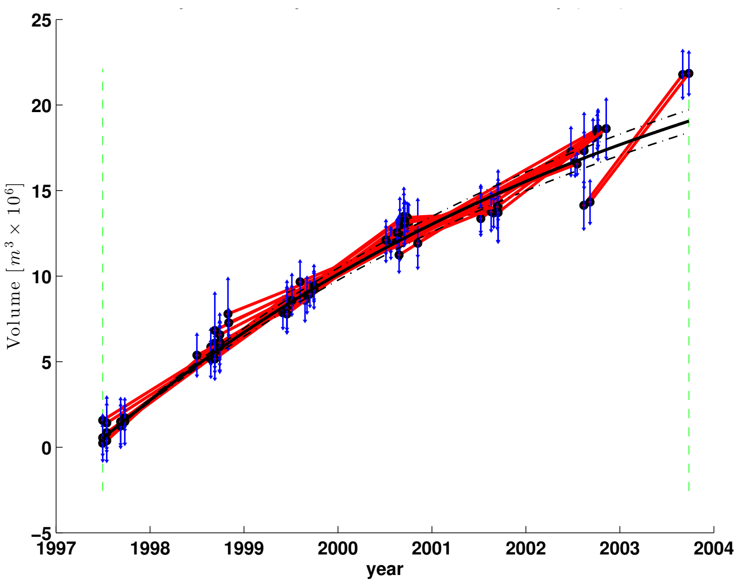

Online Resource 4. Volume increase as a function of time as estimated by temporal adjustment using the parametrization in terms of an exponentially decaying rate. The characteristic time scale is $\tau = 6.5$ years and reference time epoch at May 23, 1997. Misfit $\sigma_0 = 0.9455$. Plotting conventions as in Figures 6 through 10.
